# The Jamaica Salt Consumption Study Protocol: Sodium Intake; Sodium Content in Restaurant Foods; Knowledge, Attitudes, and Practices; Spot Urine Sodium Validation

**DOI:** 10.12688/f1000research.122619.2

**Published:** 2023-11-24

**Authors:** Trevor S. Ferguson, Karen Webster-Kerr, Marshall K. Tulloch-Reid, Nadia R. Bennett, James Ho, Tamu Davidson, Andriene Grant, Kelly-Ann Gordon-Johnson, Ishtar Govia, Suzanne Soares-Wynter, Novie Younger-Coleman, Joette McKenzie, Evelyn Walker, Simon Anderson, Sharmaine Edwards, Simone Spence

**Affiliations:** 1 Epidemiology Research Unit, Caribbean Institute for Health Research, The University of the West Indies, Mona, Kingston 7, Jamaica; 2 Ministry of Health and Wellness, Kingston 5, Jamaica; 3 Chronic Disease and Injury Department, Surveillance, Disease Prevention & Control Division, Caribbean Public Health Agency, Port of Spain, Trinidad and Tobago; 4 Caribbean Regional Office, Centers for Disease Control and Prevention, United States Embassy, Kingston 6, Jamaica; 5 Tropical Metabolism Research Unit, Caribbean Institute for Health Research, The University of the West Indies, Mona, Kingston 7, Jamaica; 6 George Alleyne Chronic Disease Research Centre, Caribbean Institute for Health Research, The University of the West Indies, Cave Hill, Bridgetown, St. Michael, BB11115, Barbados

**Keywords:** Jamaica, salt intake, sodium content, urinary sodium, salt reduction strategies, knowledge attitudes and practices, restaurant foods

## Abstract

**Background:**

Excess dietary salt consumption is a major contributor to hypertension and cardiovascular disease. Public education programs on the dangers of high salt intake, and population level interventions to reduce the salt content in foods are possible strategies to address this problem. In Jamaica, there are limited data on the levels of salt consumption and the population’s knowledge and practices with regards to salt consumption. This study therefore aims to obtain baseline data on salt consumption, salt content in foods sold in restaurants, and evaluate knowledge, attitudes, and practices of Jamaicans regarding salt consumption.

**Methods:**

The study is divided into four components. Component 1 will be a secondary analysis of data on urinary sodium from spot urine samples collected as part of a national survey, the Jamaica Health and Lifestyle Survey 2016-2017. Component 2 will be a survey of chain and non-chain restaurants in Jamaica, to estimate the sodium content of foods sold in restaurants. Component 3 is another national survey, this time on a sample 1,200 individuals to obtain data on knowledge, attitudes and practices regarding salt consumption and estimation of urinary sodium excretion. Component 4 is a validation study to assess the level of agreement between spot urine sodium estimates and 24-hour urinary sodium from 120 individuals from Component 3.

**Discussion:**

This study will provide important baseline data on salt consumption in Jamaica and will fulfil the first components of the World Health Organization SHAKE Technical Package for Salt Reduction. The findings will serve as a guide to Jamaica’s Ministry of Health and Wellness in the development of a national salt reduction program. Findings will also inform interventions to promote individual and population level sodium reduction strategies as the country seeks to achieve the national target of a 30% reduction in salt consumption by 2025.

## Introduction

High blood pressure is the leading risk factor for the global burden of disease and is responsible for over nine million deaths worldwide each year. ^1^^,^^2^ In Jamaica, data from the Jamaica Health and Lifestyle Survey 2016-2017 (JHLS-III) showed that two-thirds of the population have an abnormally high blood pressure; approximately one third (33.8%) had hypertension (more than 140/90 mmHg) and 34.0% had prehypertension (120-139 mmHg systolic or 80-89 mmHg diastolic) using the JNC-7 classification. ^3^ Data from the Statistical Institute of Jamaica (STATIN) revealed that cardiovascular disease (CVD) was responsible for almost 6,000 deaths in 2016, representing 32% of all deaths. ^4^ Given the high burden of hypertension and the fact that hypertension is responsible for approximately 50% of CVD deaths, ^5^ it is imperative that the Jamaican public be aware of the adverse effects of hypertension and its risk factors. In addition, Jamaica’s public health agencies and non-governmental organizations working in the health sector have a responsibility to implement programs to mitigate the impact of elevated blood pressure on population health.

There are many factors that contribute to the burden of hypertension, including salt consumption, obesity, insulin resistance, psychosocial stress, and genetic predisposition. There are also associations with smoking and harmful use of alcohol. Reducing dietary salt consumption, reducing obesity, smoking cessation, and avoiding harmful use of alcohol are among the strategies that can be implemented to reduce hypertension and CVD at the population level.

At present, there is strong evidence to support the potential impact of dietary salt reduction in the general population as a strategy for the reduction of hypertension and CVD. ^6^^–^^12^ For example, the United Kingdom (UK) developed and successfully implemented a program of voluntary salt reduction in collaboration with the food industry in the early 2000s, in which mean population salt intake was reduced by 15%, from 9.5 g daily in 2003 to 8.1 g daily in 2011. ^8^^,^^9^ Over the same period, there was a corresponding reduction in population mean systolic blood pressure (SBP) of 2.7 mmHg after adjusting for potential confounding factors. ^8^^,^^9^ Stroke and ischemic heart disease (IHD) mortality were reduced by 36% over the same period; it was estimated that approximately 30% of the reduction in stroke and 20% of the reduction in IHD were attributable to the reduction in salt intake. ^8^

The population approach as a strategy to achieve sodium reduction is supported by recommendations from the World Health Organization (WHO) and Pan American Health Organization (PAHO). ^2^^,^^13^ Key components of the strategy recommended by WHO and PAHO include public education programs, standardized food labelling, gradual reduction of salt content in processed foods and national surveillance systems to identify salt intake levels and major sources of salt intake. It is widely believed that population-based approaches are required to achieve the desired salt reduction, as most dietary salt consumption comes from processed foods and foods prepared outside the home, rather than by adding salt at the table. ^2^^,^^13^

In addition to the population-based strategies mentioned above, several studies have shown that individual-level and community-based interventions, including salt substitution, education on salt reduction strategies, use of digital platforms and electronic education materials are effective in reducing blood pressure and cardiovascular disease. ^14^^–^^17^ Of note, Aliasgharzadeh *et al*. found that multicomponent interventions may be more effective than single component interventions. ^16^

Measurement of sodium intake in populations is challenging. Measures include dietary assessment and measurement of urinary sodium excretion. ^18^ Dietary sodium assessment requires significant time commitments, often underestimates sodium consumption, and is considered by some to be unsuitable for measuring population sodium intake. ^18^ Currently, 24-hour urinary sodium excretion is considered the gold standard method for assessment of sodium consumption. ^18^^–^^20^ This procedure is appropriate, given that approximately 90% of consumed sodium is excreted in the urine. ^18^ The 24-hour urine collection is still challenging to accomplish in large population-based studies, resulting in some studies estimating 24-hour urine excretion from a spot urine specimen. ^18^^–^^20^ A number of formulae have been published for estimating 24 hour urine sodium excretion from spot urine specimens, including the Tanaka formula, ^21^ the Kawasaki formula ^22^ the INTERSALT formula, ^23^ and the PAHO formula. ^13^ A recent systematic review and meta-analysis found that estimates of mean population sodium intake from spot urine samples can provide a good indication of overall population salt intake with excellent sensitivity and specificity. ^20^

Globally, salt consumption is much higher than recommended. In 2010, global mean sodium intake was 3.95 g/day, with intake in men being approximately 10% higher than in women. ^24^ Intakes were highest in East Asia, Central Asia and Eastern Europe and lowest in sub-Saharan Africa and Latin America. ^24^ In the United States mean sodium intake was approximately 3.6 g/day and exceeded the recommended daily intake of 2.3 g/day for all age groups except for those 2-3 years old. ^25^ Data from the Health of the Nation Study in Barbados showed that mean sodium intake was 2.7 g/day, with intakes higher in men and young people. ^26^ Approximately 67% of individuals exceeded the WHO recommended limit of 2.0 g/day. ^26^

There are very little data on salt consumption patterns among Jamaicans, but it is generally believed that salt consumption is high. This is due to frequent consumption of salted meats ( *e.g.*, salt fish, salt mackerel, salted pork) in popular local dishes, frequent consumption of canned foods (such as corned beef), and frequent intake of fast foods, which are often high in salt content. Data from the 1990s provide some evidence for high salt consumption in Jamaica. In the Sodium Reduction Trial (SORT), baseline sodium excretion was found to be 149 mEq/day (3,437 mg/day) among a sample of 56 patients. ^27^ Additionally, the Spanish Cohort Study reported estimated sodium excretion of 3.3 g/24 hour, ^28^ but no recent or national estimates are available. More recently, data from JHLS-III found that among urban participants only 52% were classified as having a low salt diet, defined as no added salt at the table and infrequent consumption of processed foods. ^29^

In keeping with the recommendations of WHO and PAHO and consistent with Caribbean Community (CARICOM) recommendations and a mandate of the Ministry of Health and Wellness in Jamaica, we propose the implementation of a national salt reduction program in order to reduce blood pressure and associated CVD. The program will be aligned with the overall Ministry of Health’s National Strategic and Action Plan for Prevention and Control of Non-Communicable Diseases (NCDs) and the mandate of the Food Industry Task Force. Implementation of a national salt reduction program and achieving the desired effect of reduced blood pressure and associated CVD will require a sequence of actions including the steps included in the WHO SHAKE Technical Package for Salt reduction Program. These strategies include: (i) “measure and monitor salt intake” in the population; (ii) “promote reformulation of foods and meals to contain less salt”; (iii) “implement standards for effective and accurate labelling and marketing of food”; (iv) “educate and communicate to empower individuals to eat less salt”; and (v) creating “support settings to promote healthy eating”. ^2^

Jamaica has adopted the global target set by WHO to reduce salt consumption by 30% by 2025. However, Jamaica does not have any baseline data on current salt consumption and therefore would be unable to indicate whether these targets are being met. Additionally, Jamaica does not any available data on salt content in restaurant foods, but a previous study had collected data on sodium content in packaged foods. ^30^

The objectives of this study are as follows:

1. **Objective 1:** To estimate dietary sodium consumption among Jamaicans using spot urinary sodium analyses.
2. **Objective 2:** To assess the use of sodium in the preparation of restaurant foods and estimate the sodium content in restaurant foods.
3. **Objective 3:** To conduct a baseline survey on current knowledge, attitudes, and practices regarding salt intake in Jamaica, and estimate current levels of salt consumption. We will also evaluate associations between knowledge and attitudes regarding salt consumption and actual estimated salt consumption, dietary and other health behaviors, and health characteristics including, blood pressure and body mass index.
4. **Objective 4:** To evaluate the accuracy of spot urine sodium as a measure of dietary sodium intake in the Jamaican setting by comparing estimates to 24-hour urinary sodium excretion.

Further details on data sources, sample size and procedures are given in the Methods section.

## Methods

The project will consist of the following components in order to fulfil objectives 1-4 outlined in the Introduction:

1. **Component 1**: A cross-sectional analysis of data from approximately 1,000 spot urine samples collected as part of the JHLS-III.
2. **Component 2**: A baseline survey of sodium content in commonly consumed foods sold in restaurants chains and individual restaurants.
3. **Component 3:** Conducting a baseline survey of knowledge, attitudes and practices regarding salt consumption and estimation of urinary sodium excretion, dietary and other health behaviors, blood pressure and anthropometry.
4. **Component 4:** Validation study assessing agreement between spot urinary sodium and 24-hour urinary sodium in assessing dietary salt consumption.

Details of each component are described below.

### Component 1: spot urine sodium analyses

*Data sources*

Data from 1,091 spot urine sodium samples, along with relevant sociodemographic and biomedical data were obtained from the third Jamaica Health and Lifestyle Survey (JHLS-III), which was conducted between September 2016 and February 2017. ^3^ Some details on the procedures used in JHLS-III have been published. ^29^ The survey enrolled a nationally representative sample of Jamaicans 15 years and older using a multi-stage sampling design. In the first stage, 171 enumeration districts (EDs) were randomly selected from a national total of 6,241 EDs, with selection probability proportionate to the size of the ED. In the second stage, 20 households (dwellings) are systematically selected within each ED, using a random starting point. Within each household one individual is selected using the Kish method. ^31^ The survey collected data on a wide cross-section of health issues using questionnaires, physical measurements, point-of-care and laboratory measurements and geo-informatics (GIS) mapping. An early morning spot urine sample was collected from study participants and urine sodium measured using the Roche 9180 Electrolyte Analyzer (Mannheim, Germany). These data along with relevant sociodemographic data and data on hypertension and risk factors or comorbidities were obtained from the JHLS-III Research Group. The specific variables included are available as Extended Data. JHLS-III previously received ethical approval from the UWI (Mona) Ethics Committee (ECP 25, 15/16) and the Ministry of Health Ethics Committee (Study Number 2015/51).

*Inclusion and exclusion criteria*

For this component we will include participants from JHLS-III who have available data for urinary sodium. We will exclude participants with missing data on urinary sodium, age, or sex.

*Sample size and power*

Given that we have a fixed available sample size we estimated the power for the 1,091 available urine sodium samples. A design effect for the survey was calculated given the multistage sampling procedure. This was done by calculating the ratio of the variance of the urinary sodium values in samples from JHLS-III with and without accounting for survey design. The estimated design effect was 1.94. Using this design effect our sample of 1,091 participants translates to an effective sample size of 563 participants. Hypothesized mean sodium excretion was obtained from the Health of the Nations Study in Barbados. ^26^ Mean sodium intake in that study was 2,656 mg per day. Using the ‘ *power onemean’* command in Stata (RRID:SCR_012763) and 10% margin of error ( *i.e.*, 2,656 vs. 2,390), the effective sample size of 563 gave a power of 97.5%.

*Statistical analyses*

From these data we will estimate mean dietary sodium consumption and the proportion of patients with high dietary sodium consumption. The PAHO formula will be used to estimate 24-hour urine sodium and potassium. ^23^

The PAHO formula uses measured spot urine sodium or potassium divided by measured spot urine creatinine multiplied by estimated 24-hour urine creatinine as shown below. ^32^^,^^33^ The 24-hour urine creatinine was estimated using the Chronic Kidney Disease Epidemiology Collaboration (CKD-EPI) formula. ^32^^,^^33^

Equation 1. PAHO formula for 24-hour urine sodium.

$$\left[\text{Estimated}\;24-\text{hour value}=\left(\text{measured spot urine}\;\mathrm{Na}\;\text{or}\;K÷\text{measured spot urine}\;\mathrm{Cr}\right)\times \text{estimated}\;24-\text{hour urinary}\;\mathrm{Cr}\right].$$

Equation 2. CKD-EPI formula for 24-hour urine creatinine.

$$\left[\text{Estimated}\;24-\text{hour urine}\;\mathrm{Cr}=879.89+12.51\times \text{weight}\;\left(\mathrm{kg}\right)-6.19\times \mathrm{age}+\left(34.51\;\text{if black}\right)-\left(379.42\;\text{if female}\right)\right]$$

Note: PAHO, Pan American Health organization; CKD-EPI, Chronic Kidney Disease Epidemiology Collaboration; Cr, creatinine; BMI, body mass index; Na, sodium; K, potassium.

We will also estimate how dietary sodium consumption vary by sex and other sociodemographic factors, self-reported dietary practices, and biomedical characteristics, including blood pressure levels and hypertension awareness. Multivariable regression models will be used to identify factors associated with high sodium intake. Analyses will be weighted to account for survey design.

### Component 2: salt content of foods sold in restaurants

For this component, the research team will conduct a search to obtain a listing of registered food establishments (defined as serving pre-prepared meals) within Jamaica. All restaurants serving meals that are ready to eat will be eligible for inclusion. A list of such food outlets will be obtained by reviewing listings from the Jamaica Telephone Directory and contacting relevant regulatory organizations responsible for licensing food establishments under Jamaica’s Public Health Act. After compiling the list of food establishments, we will select a sample consisting firstly of restaurant chains, defined as having three or more local branches, then a sample of other restaurants with one or two local branches. This sample of non-chain food establishments will comprise five randomly selected restaurants/cook shops for each of the 60 electoral districts selected for ‘ *Component 3*’ of the study as described below. This will result in a sample of 300 non-chain restaurants. The number of chain restaurants will be determined after we have completed our search for registered food establishments. There is no standard system of restaurant classification in Jamaica. In our analyses we will further define restaurants as fast food restaurants, standard dining in restaurants, and cafes.

Inclusion criteria for this phase of the study will be food establishments that sell ready to eat food items prepared at the site of the restaurant or cookshop. Establishments that sell only packaged food prepared elsewhere will be excluded.

For each selected food establishment, we will contact the restaurant owner or manager initially *via* telephone and introduce the study and its objectives. The consent form for food establishments can be found as *Extended data.* ^41^ If necessary, an in-person meeting will be arranged. We will seek to obtain a listing of meal items sold in these establishments and information on recipes/ingredient quantities, methods of food preparation and portion sizes to estimate salt content of foods sold by the establishment. We will also seek to obtain information on nutrient content of food sold if these have been previously determined. We will also gather publicly available data on recipes, ingredient quantities, and salt content of meals from restaurant websites and/or social media pages. Where we have difficulty obtaining specific recipes or ingredient quantities, we will estimate salt content based on typical recipes for similar items in the Jamaican context. We will also seek to obtain data from sources such as the US Department of Agriculture Food Central Database international chain restaurants. We will document the extent to which our estimates are based on shared recipes and reported methods of preparation as opposed to estimates made from typical recipes. Foods will again be categorized into groups ( *e.g.*, burgers, fries, chicken preparations, fish preparations, patties, loaves, cooked lunches). The specific food categories with be decided in the analysis stage of the study, guided by the study nutritionist. For each food group, we will estimate mean sodium content per meal and proportion of foods with high salt content. Given that analyses will be purely descriptive, we have not performed sample size or power calculations, but we will report findings based on the available data. We will, however, compute power after the restaurants have been selected in order to assess the probable type 2 error associated with our estimates.

### Component 3: survey of knowledge, attitudes and practice regarding salt intake and estimation of salt consumption levels

The survey will use a nationally representative sample of 1,200 participants aged 18 years or older. We will approach STATIN to select a total of 60 EDs from all 14 parishes, using probability proportionate to size. Within each ED, we will select 20 households and within each household we will select one individual, using the Kish method. ^31^

*Inclusion and exclusion criteria*

We will include non-institutionalized adults, aged 18 years and older, resident in the selected ED. We will exclude institutionalized participants or individuals who are unable to complete the questionnaire due to impaired capacity to understand the questions asked or inability to speak. No other health or social condition were considered as inclusion or exclusion criteria. However, we are collecting data on participants use of medications for hypertension and, specifically, if they are using diuretics. We will assess whether there are differences in urine sodium excretion for those with hypertension or on diuretic therapy. Where necessary we will exclude participants on diuretic therapy from analyses or conduct sensitivity analyses with models excluding and including persons on diuretic therapy.

*Recruitment*

For each selected community we will first obtain a map of the community from STATIN and then arrange a visit to the community to establish the number of households and determine the household sampling interval. We aim to select 20 individuals from each ED, one from each household. The household sampling interval will therefore be determined by dividing the number of households by 20. A visit will be made to each selected household and one person will be selected using the Kish method. ^31^ For this procedure, the trained data collectors make a list of all the household members who are eligible to participate in the study, ordered by age and sex and the select one person based on a random number system. If the participant from the selected household refuses to participate we will attempt to recruit a participant from the neighboring household. Recruitment will be supervised by the study’s project coordinator.

*Data collection*

For each selected participant, we will first obtain informed consent (verbal or written) and then administer a questionnaire to evaluate knowledge about salt intake and health, attitudes with regards to salt intake and low salt diet, and current practices of salt intake. Where verbal consent is obtained this would be witnessed and signed by the research assistant obtaining consent. This informed consent procedure has been approved by the UWI and the Ministry of Health and Wellness Ethics Committees. The questionnaire and consent form can be found as *Extended data.* ^39^^,^^40^ The questionnaire was developed based on a review of published questionnaires on dietary salt intake identified through a search of PubMed (RRID:SCR_004846) and Google (RRID:SCR_017097). ^34^^–^^36^ Questions were adapted to ensure cultural appropriateness for the Jamaican context.

In addition to the questions on knowledge attitudes and practices regarding salt intake, we will also administer a partial food frequency questionnaire (FFQ) to assess frequency of consumption of high salt foods and compare this with the frequency of consumption of low salt or other healthy foods. The FFQ was derived from a local FFQ which was previously validated for use in Jamaica. ^37^ The FFQ has undergone several updates using 24-hour recalls to calibrate food listings and capture detailed use of sodium and related food preparation practices. Instruments have been used successfully in a sodium reduction trial (Jamaica) and a more recent Barbados study of ultra-processed food (UPF) intakes. ^27^^,^^38^

Additional questionnaire items will include physical activity and smoking practices as measures of other health behaviors. Physical activity will be assessed using the English version of the Short International Physical Activity Questionnaire (IPAQ), which is now in the public domain. The full questionnaire (including sections on knowledge, attitudes and practices, food frequency and physical activity) was pre-tested to ensure accuracy and acceptability within the Jamaican context. Pre-testing involved the administration of the questionnaire to approximately 20 individuals who fit the project’s inclusion criteria and assess time taken to administer the questionnaire, making note of any difficulty with answering specific questionnaire items and ask for specific feedback on the questionnaire. Adjustments were then made to improve understandability and acceptability. We will also measure weight, height, waist circumference, hip circumference and blood pressure and collect an early morning, or casual, spot urine sample to measure urine sodium excretion. Instructions for collecting the urine sample will be given at the time of recruitment into the study. Urine sodium will be measured using the Roche 9180 Electrolyte Analyzer (Mannheim, Germany) at the Tropical Metabolism Research Unit laboratory.

*Coronavirus disease 2019 (COVID-19) considerations*

Field work for this component began in the first quarter of 2022. Interviews for questionnaire administration are completed *via* telephone to minimize person to person contact. In light of the COVID-19 pandemic we have instituted contact precautions in keeping with the Mona Research Ethics Committee Guidelines on conducting face-to-face research in light of COVID-19. Specifically, we will require all data collectors to wear masks and practice appropriate hand sanitization and handwashing during recruitment and measurement. Respondents will also be required to wear masks during the interactions with data collectors. We will provide disposable masks for respondents where necessary. All data collectors are encouraged to receive the COVID-19 vaccine prior to the beginning of data collection.

*Sample size and power*

Sample size was calculated using the *‘power onemean’* command in Stata. Using the mean of 2,656 mg/day from the Barbados Health of the Nation Study as outlined above ^26^ and a 7.5% margin of error ( *i.e.*, 2,656 vs. 2,457), the required sample size with alpha 0.05 and power 80% was estimated as 511. Given the design effect of 1.94 as calculated above, the minimum sample size accounting for survey design would be 991. We will target a sample size of 1,200 participants (20% above the minimum sample size) to allow for participant non-response and missing data.

*Data management*

Data will be collected by trained data collectors using password-protected tablet computers, with REDCap (RRID:SCR_003445) data collection software. We will include range and consistency checks to minimize data entry errors. Data collected on the tablets will be protected using data encryption. Options to transfer data to external drives ( *e.g.*, USB flash drives and SD cards will be disabled. Data will then be uploaded to a secure server using data encryption protocols. Deidentified data will then be downloaded for analysis. Access to data will be limited only to members of the study’s data analysis team. De-identified data will be kept indefinitely. Identifiable data, such as consent forms will be kept on secure encrypted servers and will be destroyed by permanent deletion from the server after five years. To assess data accuracy and reliability of data, we will have project staff recollect data from 5–10% of respondents to check against the original collected data.

*Statistical analyses*

From these data we will estimate the proportion of participants reporting appropriate knowledge on the effects of salt on health, and the proportion of participants with various attitudes and practices regarding salt intake. We will also obtain mean urine sodium concentration and estimate 24-hour urine sodium using the PAHO (Equation 1) equations. We will also explore the development of a new equation for use in Jamaica and similar populations from our validation study in ‘ *Component 4*’, outlined below. We will then estimate the proportion of patients with high dietary sodium consumption. We will also evaluate how dietary sodium consumption, knowledge, attitudes and practices vary by sex, other sociodemographic factors, and biomedical characteristics, including blood pressure levels and hypertension awareness. The analyses for the evaluation of knowledge attitudes and practices will be primarily descriptive, but we will use scores to classify persons in KAP categories. These categories will be created in the analysis phase of the study. Multivariable regression models will be used to identify factors associated with high sodium intake and with good knowledge, attitudes, or practices related to salt intake. Analyses will be weighted to account for survey design.

### Component 4: validation study using 24-hour urinary sodium

A 10% sub-sample from ‘ *Component 3*’, described above, will be asked to provide a 24-hour urine sample to measure 24-hour urine sodium excretion to assess the accuracy of spot urine sodium estimation and the correlation between spot urinary sodium and 24-hour urinary sodium. The sub-sample will consist of participants from the first six EDs to be recruited for the survey in ‘ *Component 3*’. All participants from these first six EDs will be included. We will exclude participants who are unwilling to carry out the 24-hour urine collection. If we have less than 120 participants from the first six EDs, we will continue to recruit from the next EDs until the targeted sample of 120 individuals is reached. We will estimate 24-hour urine sodium from the spot urine sodium and then assess the levels of agreement between 24-hour urine sodium estimated from spot urinary sodium and 24-hour urinary sodium from the 24-hour urine sample. Data from this validation study will be used to calibrate equations for estimating 24-hour urinary sodium from spot urinary sodium measurements. These new equations from local data may then be used for this and future studies on urine sodium excretion.

The procedure for collecting 24-hour urine sample will be as follows: On the morning of the start of the 24-hour period, the participant must urinate to completely empty the bladder and record the start time. This “first-pass urine” is discarded. Subsequently all urine passed is collected in a container, including the first urine of the following morning. This should be at the same time as the previous morning to ensure 24 hours is completed. The time the final urine sample is passed is recorded. The container with the 24-hour urine sample will be collected from the participant later that morning. The information sheet for the 24-hour urine collection can be found as *Extended data.* ^42^

After the samples have been collected, we will assess we will assess urine volume, measure urine sodium concentration, urine potassium concentration and urine creatinine. We will exclude participants whose 24 hour urine volume is <500 ml or collection time is <22 hours; we will also exclude participants whose 24 hr urine creatinine is greater than 2SD above or below the sex-specific mean (Du et al 2021). Where collection time is shorter or longer than 24 hours, we will adjust the volume to 24-hours by dividing the total volume by collection time (in hours) and multiplying by 24 (Jackson et al 2020).

*Sample size and power*

Sample size calculations were estimated using the *‘power onecorrelation’* command in Stata. Estimated correlation coefficients for spot urinary sodium compared to 24-hour urinary sodium were obtained from Brown *et al.*, ^23^ and Tanaka *et al.*, ^21^ using data from Western and Japanese participants in the INTERSALT study. Estimated correlation coefficients were 0.46 for men and 0.34 for women in the study by Brown *et al.*, and 0.65 for men and women combined in the study by Tanaka *et al*. Using 0.40 and 0.65 as the null and alternate estimates, yield as sample size of 67. If we went for a slightly more precise estimate of 0.4 and 0.60, the required sample size would be 112 participants. The targeted 120 participants should be adequately powered to assess correlations between spot and 24-hour urine sodium in this study.

*Statistical analyses*

Agreement between different measures of dietary sodium consumption will be computed using the intra-class correlation coefficients and Bland-Altman plots. We will assess whether physical activity level is associated with 24 hour or spot urine sodium and adjust accordingly. We will also classify participants as high salt consumption based on WHO recommended values and assess the level of agreement between classification based on the spot urine sodium and 24-hour urine sodium using the kappa statistics.

### Reports and publication

The findings from the study will be shared initially as a project report to the funding agency and shared with relevant stakeholders such as the Ministry of Health and Wellness. We will also host a project dissemination symposium to share findings in a public forum. Additionally, findings from the study will be shared at academic conferences and in peer reviewed publications.

### Ethical considerations

The study will be conducted in full compliance with international ethical standards as well as the Mona Campus Research Ethics Committee and Ministry of Health Advisory Panel on Ethics and Medico-Legal Affairs guidelines for the conduct of research. Ethical approval for the study was obtained from the University of the West Indies Mona Campus Research Ethics Committee (study number: CRECMN.153 2020/2021; approved August 17, 2021) and the Ministry of Health and Wellness Advisory Panel on Ethics and Medico-Legal Affairs (study number: 2021/05; approved August 25, 2021).

This is a minimal risk study, involving analyses of previously collected data, questionnaires on non-sensitive subjects and non-invasive measurements. Participants will provide written informed consent or witnessed verbal consent, given the limitation of face-to-face contact due to COVID-19. Where face-to-face contact is necessary, we will observe appropriate precautions, including wearing of masks and hand sanitization. Data will be stored on password-protected computers or tablets, or in locked filing cabinets, and treated with strict confidentiality. Each study participant will be given a unique study identification number that will be recorded on the consent forms and data collection records. Consent forms will be kept separately from the data collection records to protect participants identity. There are no direct benefits to the participants, except for receiving information on blood pressure, body size, and the effects of salt on health. Participants will be selected based on their residence in randomly selected communities and not because of easy availability or diminished autonomy. The restaurant owners or managers will also provide written or verbal informed consent.

### Study status

At present we are conducting data analyses to complete the requirements for *‘Component 1’*, creating a database of restaurants in Jamaica for *‘Component 2’* and have begun field work and data collection for *‘Component 3’* and *‘Component 4’.*

## Discussion

This study will provide important baseline data on salt consumption in Jamaica and will fulfil interventions recommended in the first component of the WHO SHAKE Technical Package for Salt Reduction. The findings will serve as a guide to Jamaica’s Ministry of Health and Wellness in the development of a national salt reduction program. Findings will also inform interventions to promote individual and population level sodium reduction strategies as the country seeks to achieve the national target of a 30% reduction in salt consumption by 2025.

This study has some limitations. Given the limited funds available for the study, we have constrained the sample size of ‘ *Component 3*’ to 1,200 individuals. While this will provide reasonable power for our main study question, estimates for subgroups with the population may be imprecise and our ability to identify associations between salt intake and some characteristics may be limited. We also anticipate that some restaurant owners may be unwilling to share information on ingredient quantities/recipes and as such some of these estimates may be based on usual practice by our nutritionist. Additionally, it would have been useful to measure salt content in food samples for the restaurants, but we do not have funding for this. We intend to seek funding to perform such measurements in future studies. For component 4, we did not collect information on lean meat intake and therefore will not be able to assess how this relates to 24-hour urine sodium.

The strengths of this study include the use of a nationally representative population-based sample, thus ensuring that the findings will be generalizable to the Jamaican population. The multi-component structure of this study will ensure that we address several important questions related to salt and health in the Jamaican context. The broad team on investigators and the joint effort between the Ministry of Health and Wellness and the University of the West Indies will ensure that the findings are taken up and translated into policy.

Overall, we believe that this is an important study for Jamaica with the potential to have a major impact on public health policy. Findings will also be relevant and applicable to other countries in the Caribbean region and other populations with similar socio-cultural characteristics, including black populations in North America and Europe. The study therefore has the capacity to improve the lives of people in Jamaica and beyond.

## Data availability

### Underlying data

No data are associated with this article.

Data for the Jamaica Health and Lifestyle Survey 2016-2017, ^3^ which are being used in this study may be accessed by contacting the investigators at caihr@uwimona.edu.jm.

### Extended data

Figshare: The Jamaica Salt Consumption, Knowledge, Attitudes and Practices (Salt-KAP) Study Participant Questionnaire. https://doi.org/10.6084/m9.figshare.20056850. ^39^

Figshare: Consent form for individual participants in the Jamaica Salt Consumption, Knowledge, Attitudes and Practices (Salt-KAP) Study. https://doi.org/10.6084/m9.figshare.20057189. ^40^

Figshare: Consent form for food establishments for the Jamaica Salt Consumption, Knowledge, Attitudes and Practice (Salt-KAP) Study. https://doi.org/10.6084/m9.figshare.20057204. ^41^

Figshare: 24 Hour Urine Collection Information Sheet for Salt-KAP Study. https://doi.org/10.6084/m9.figshare.20129903. ^42^

Figshare: Data to be Obtained From JHLS-III For Component 1 of the Jamaica Salt Consumption Study. https://doi.org/10.6084/m9.figshare.24589452. ^43^

Data are available under the terms of the Creative Commons Attribution 4.0 International license (CC-BY 4.0).

## References

[1] Lim SS Vos T Flaxman AD : A comparative risk assessment of burden of disease and injury attributable to 67 risk factors and risk factor clusters in 21 regions, 1990-2010: a systematic analysis for the Global Burden of Disease Study 2010. *Lancet.* 2012;380(9859):2224–2260. 10.1016/S0140-6736(12)61766-8 23245609 PMC4156511

[2] World Health Organization: *The SHAKE Technical Package for Salt Reduction.* Geneva, Switzerland: World Health Organization;2016.

[3] Jamaica Health and Lifestyle Survey III Investigators: Jamaica Health and Lifestyle Survey III (2016-2017) Preliminary Key Findings. 2018.

[4] Statistical Institute of Jamaica: *Demographic Statistics 2017.* Kingston, Jamaica: Statistical Institute of Jamaica;2018.

[5] World Health Organization: *A global brief on hypertension.* Geneva, Switzerland: WHO Press;2013. Reference Source

[6] Bibbins-Domingo K Chertow GM Coxson PG : Projected effect of dietary salt reductions on future cardiovascular disease. *N. Engl. J. Med.* 2010;362(7):590–599. 10.1056/NEJMoa0907355 20089957 PMC3066566

[7] Graudal NA Hubeck-Graudal T Jurgens G : Effects of low sodium diet versus high sodium diet on blood pressure, renin, aldosterone, catecholamines, cholesterol, and triglyceride. *Cochrane Database Syst. Rev.* 2017;4:Cd004022. 10.1002/14651858.CD004022.pub4 28391629 PMC6478144

[8] He FJ MacGregor GA : Role of salt intake in prevention of cardiovascular disease: controversies and challenges. *Nat. Rev. Cardiol.* 2018;15(6):371–377. 10.1038/s41569-018-0004-1 29713009

[9] He FJ Pombo-Rodrigues S Macgregor GA : Salt reduction in England from 2003 to 2011: its relationship to blood pressure, stroke and ischaemic heart disease mortality. *BMJ Open.* 2014;4(4):e004549. 10.1136/bmjopen-2013-004549 24732242 PMC3987732

[10] Hyseni L Elliot-Green A Lloyd-Williams F : Systematic review of dietary salt reduction policies: Evidence for an effectiveness hierarchy? *PLoS One.* 2017;12(5):e0177535. 10.1371/journal.pone.0177535 28542317 PMC5436672

[11] Sutherland J Edwards P Shankar B : Fewer adults add salt at the table after initiation of a national salt campaign in the UK: a repeated cross-sectional analysis. *Br. J. Nutr.* 2013;110(3):552–558. 10.1017/S0007114512005430 23286885

[12] Wyness LA Butriss JL Stanner SA : Reducing the population's sodium intake: the UK Food Standards Agency's salt reduction programme. *Public Health Nutr.* 2012;15(2):254–261. 10.1017/S1368980011000966 21729460

[13] Pan American Health Organization: Preventing Cardiovascular Disease in the Americas by Reducing Dietary Salt Intake Population-Wide.2009.

[14] Yin X Rodgers A Perkovic A : Effects of salt substitutes on clinical outcomes: a systematic review and meta-analysis. *Heart.* 2022;108(20):1608–1615. 10.1136/heartjnl-2022-321332 35945000

[15] Brand A Visser ME Schoonees A : Replacing salt with low-sodium salt substitutes (LSSS) for cardiovascular health in adults, children and pregnant women. *Cochrane Database Syst Rev.* 2022;8. 10.1002/14651858.CD015207 35944931 PMC9363242

[16] Aliasgharzadeh S Tabrizi JS Nikniaz L : Effect of salt reduction interventions in lowering blood pressure: A comprehensive systematic review and meta-analysis of controlled clinical trials. PLoS One. 2022;17(12): e0277929. 10.1371/journal.pone.0277929 36477548 PMC9728935

[17] Jarrar AH Al Dhaheri AS Lightowler H : Using Digital Platform Approach to Reduce Salt Intake in a Sample of UAE Population: An Intervention Study. Front. Public Health. 2022;10: 860835. 10.3389/fpubh.2022.860835 35685760 PMC9172248

[18] McLean RM : Measuring Population Sodium Intake: A Review of Methods. *Nutrients.* 2014;6(11):4651–4662. 10.3390/nu6114651 25353661 PMC4245554

[19] He FJ Ma Y Campbell NRC : Formulas to Estimate Dietary Sodium Intake From Spot Urine Alter Sodium-Mortality Relationship. *Hypertension.* 2019;74(3):572–580. 10.1161/HYPERTENSIONAHA.119.13117 31352828

[20] Huang L Crino M Wu JH : Mean population salt intake estimated from 24-h urine samples and spot urine samples: a systematic review and meta-analysis. *Int. J. Epidemiol.* 2016;45(1):239–250. 10.1093/ije/dyv313 26796216

[21] Tanaka T Okamura T Miura K : A simple method to estimate populational 24-h urinary sodium and potassium excretion using a casual urine specimen. *J. Hum. Hypertens.* 2002;16(2):97–103. 10.1038/sj.jhh.1001307 11850766

[22] Kawasaki T Itoh K Uezono K : A simple method for estimating 24 h urinary sodium and potassium excretion from second morning voiding urine specimen in adults. *Clin. Exp. Pharmacol. Physiol.* 1993;20(1):7–14. 10.1111/j.1440-1681.1993.tb01496.x 8432042

[23] Brown IJ Dyer AR Chan Q : Estimating 24-hour urinary sodium excretion from casual urinary sodium concentrations in Western populations: the INTERSALT study. *Am. J. Epidemiol.* 2013;177(11):1180–1192. 10.1093/aje/kwt066 23673246 PMC3664342

[24] Powles J Fahimi S Micha R : Global, regional and national sodium intakes in 1990 and 2010: a systematic analysis of 24 h urinary sodium excretion and dietary surveys worldwide. *BMJ Open.* 2013;3(12):e003733. 10.1136/bmjopen-2013-003733 24366578 PMC3884590

[25] Appel LJ : Salt intake, salt restriction, and primary (essential) hypertension. *UpToDate.* Waltham, MA: Wolters Kluwer;2020 [cited January 3, 2020]. Reference Source

[26] Harris RM Rose AMC Hambleton IR : Sodium and potassium excretion in an adult Caribbean population of African descent with a high burden of cardiovascular disease. *BMC Public Health.* 2018;18(1):998. 10.1186/s12889-018-5694-0 30092782 PMC6085702

[27] Forrester T Adeyemo A Soarres-Wynter S : A randomized trial on sodium reduction in two developing countries. *J. Hum. Hypertens.* 2005;19(1):55–60. 10.1038/sj.jhh.1001782 15470483

[28] Cooper R Rotimi C Ataman S : The prevalence of hypertension in seven populations of west African origin. *Am. J. Public Health.* 1997;87(2):160–168. 10.2105/AJPH.87.2.160 9103091 PMC1380786

[29] McKenzie JA Younger NO Tulloch-Reid MK : Ideal cardiovascular health in urban Jamaica: prevalence estimates and relationship to community property value, household assets and educational attainment: a cross-sectional study. *BMJ Open.* 2020;10(12):e040664. 10.1136/bmjopen-2020-040664 33323436 PMC7745314

[30] Soares-Wynter S Aiken-Hemming SA Hollingsworth B : Applying Nutrient Profiling Systems to Packaged Foods and Drinks Sold in Jamaica. *Foods.* 2020;9(1). https://www.mdpi.com/2304-8158/9/1/65 10.3390/foods9010065 PMC7022911 31936193

[31] Kish L : A Procedure for Objective Respondent SelectionWithin the Household. *J. Am. Stat. Assoc.* 1949;44:380–387. 10.1080/01621459.1949.10483314

[32] Ix JH Wassel CL Stevens LA : Equations to Estimate Creatinine Excretion Rate: The CKD Epidemiology Collaboration. *Clin. J. Am. Soc. Nephrol.* 2011;6(1):184–191. 10.2215/CJN.05030610 20966119 PMC3022241

[33] Jędrusik P Symonides B Gaciong Z : Estimation of 24-hour urinary sodium, potassium, and creatinine excretion in patients with hypertension: can spot urine measurements replace 24-hour urine collection? *Pol. Arch. Intern. Med.* 2019;129(7-8):506–515. 10.20452/pamw.14872 31215902

[34] Cappuccio FP D'Elia L Obreja G : *Dietary Salt Intake Survey in the Republic of Moldova 2016.* Copenhagen: World Health Organization;2018.

[35] Charlton KE Steyn K Levitt NS : Development and validation of a short questionnaire to assess sodium intake. *Public Health Nutr.* 2008;11(1):83–94. 10.1017/S1368980007000146 17582243

[36] Pan American Health Organization: Questionnaire on Knowledge, Attitudes, Behavior toward Dietary Salt and Health. 2010. Reference Source

[37] Jackson MD Walker SP Younger NM : Use of a food frequency questionnaire to assess diets of Jamaican adults: validation and correlation with biomarkers. *Nutr. J.* 2011;10(1):28. 10.1186/1475-2891-10-28 21477338 PMC3094277

[38] Harris RM Rose AMC Soares-Wynter S : Ultra-processed food consumption in Barbados: evidence from a nationally representative, cross-sectional study. *J. Nutr. Sci.* 2021;10: e29. 10.1017/jns.2021.21 34094510 PMC8141678

[39] Ferguson TS Webster-Kerr K Tulloch-Reid M : The Jamaica Salt Consumption, Knowledge, Attitudes and Practices (Salt-KAP) Study Participant Questionnaire. figshare. Online resource. [Dataset]. 2022. 10.6084/m9.figshare.20056850.v1

[40] Ferguson TS Webster-Kerr K Tulloch-Reid M : Consent form for individual participants in the Jamaica Salt Consumption, Knowledge, Attitudes and Practices (Salt-KAP) Study. figshare. Online resource. [Dataset]. 2022. 10.6084/m9.figshare.20057189.v2

[41] Ferguson TS Webster-Kerr K Tulloch-Reid M : Consent form for food establishments for the Jamaica Salt Consumption, Knowledge, Attitudes and Practice (SALT-KAP) Study. figshare. Online resource. [Dataset]. 2022. 10.6084/m9.figshare.20057204.v1

[42] Ferguson TS Walker E Bennett N : 24 Hour Urine Collection Information Sheet for Salt-KAP Study. figshare. Online resource. [Dataset]. 2022. 10.6084/m9.figshare.20129903.v1

[43] Ferguson T : Data To Be Obtained From JHLS-III For Component 1 of The Jamaica Salt Consumption Study. Figshare 2023. 10.6084/m9.figshare.24589452

