# Supplementary material for: The Jamaica Salt Consumption Study Protocol: Sodium Intake; Sodium Content in Restaurant Foods; Knowledge, Attitudes, and Practices; Spot Urine Sodium Validation
Source: F1000Res. 2023 Nov 24;11:721. Originally published 2022 Jun 30. [Version 2] doi: 10.12688/f1000research.122619.2 (PMC10804057; doi:10.12688/f1000research.122619.2)
Supplement: Peer review history

# Reviewer report: Reviewer response for version 2

**DOI:** 10.5256/f1000research.159250.r233609

**Published:** 2024-01-22

**Authors:** Tânia Silva-Santos

**Affiliations:** 1 EPIUnit-Institute of Public Health, University of Porto, 4050-091, Porto, Portugal

Component 2:

 It is inaccurate to estimate the sodium in meals without analyzing the sodium content of a sample in the laboratory. They cannot estimate the added sodium.

 The procedure described allows you to identify the main sources of sodium in foods served in restaurants.

 Component 3:

 What is the procedure for assessing blood pressure and anthropometric assessment?

 What sociodemographic and health questions will they ask?

 Will all participants have a 24-hour urine collection?

 In the exclusion criteria, did you consider excluding pregnant women, patients with kidney disease and people with urinary incontinence?

Is the study design appropriate for the research question?

Yes

Is the rationale for, and objectives of, the study clearly described?

Yes

Are sufficient details of the methods provided to allow replication by others?

Partly

Are the datasets clearly presented in a useable and accessible format?

Yes

Reviewer Expertise:

Sodium intake; interventions to reduce sodium intake

I confirm that I have read this submission and believe that I have an appropriate level of expertise to confirm that it is of an acceptable scientific standard, however I have significant reservations, as outlined above.

# Reviewer report: Reviewer response for version 2

**DOI:** 10.5256/f1000research.159250.r225292

**Published:** 2023-12-11

**Authors:** Adriana Blanco-Metzler

**Affiliations:** 1 Costa Rican Institute of Research and Teaching in Nutrition and Health (INCIENSA), Tres Ríos 4-2250, Costa Rica

The manuscript was improved and its ok. Congrats.

Is the study design appropriate for the research question?

Yes

Is the rationale for, and objectives of, the study clearly described?

Yes

Are sufficient details of the methods provided to allow replication by others?

Partly

Are the datasets clearly presented in a useable and accessible format?

Yes

Reviewer Expertise:

NA

I confirm that I have read this submission and believe that I have an appropriate level of expertise to confirm that it is of an acceptable scientific standard.

# Reviewer report: Reviewer response for version 1

**DOI:** 10.5256/f1000research.134637.r176067

**Published:** 2023-08-31

**Authors:** Amjad Jarrar

**Affiliations:** 1 Department of Nutrition and Health, College of Medicine and Health Sciences, United Arab Emirates University, Al Ain, Abu Dhabi, United Arab Emirates

Very good study with clear objectives. My comments mainly related to the following points:

- Inclusion and exclusion criteria: you should include more details for exclusion criteria although you are using spot urine you need to explain if you are excluding participants with hypertension medications or diuretics medications. these points should include in exclusion even if spot urine is used. 
- Are you using two formulas to estimate sodium intakes **NTERSALT formula and PAHO formula....are aiming to compare between them or you will use one formula and which one fit Jamaica population.
- Component 2: salt content of foods sold in restaurants. Very good but the aim should be clear. the aim her not to estimate sodium but to identify main source of sodium in food intakes. 
- Component 3, how you will assess KAP which protocol or questionnaire and are you using scoring system just to describe KAP. 
- Component 4, In order to do it correct you need to include more information and measurements regarding exclusion criteria such as acceptable total 24-hour urine sample 500 ml and above, acceptable level of 24-hour creatinine to measure adequate 24-hour urine sample, measure amount of lean meat intake, these points can be found in studies dealing with 24-hour urine. Including IPAQ very good but not limited to assess physical activity but also important for justify differences in estimated sodium intake between 24-hour and spot urine. 
- General notes, what are reference for food frequency questionnaire is it general or specific for salt intake (Data collection).
- it will be good to include intervention studies in references and introduction to highlight benefit of this study for future intervention studies. 

Is the study design appropriate for the research question?

Yes

Is the rationale for, and objectives of, the study clearly described?

Partly

Are sufficient details of the methods provided to allow replication by others?

Partly

Are the datasets clearly presented in a useable and accessible format?

Yes

Reviewer Expertise:

Knowledge Attitude and Practice toward salt intake, Sport Nutrition and Non-communicable diseases. Glycemic index and Glycemic load.

I confirm that I have read this submission and believe that I have an appropriate level of expertise to confirm that it is of an acceptable scientific standard, however I have significant reservations, as outlined above.

# Response

**Published:** 2023-11-22

**Authors:** Trevor S Ferguson

**Affiliations:** The University of the West Indies, Jamaica

To: Amjad Jarrar

 Thank you for taking the time to review this manuscript and providing insightful suggestions. 

 Responses to the specific comments are given below. Responses are in bold type.

- Inclusion and exclusion criteria: you should include more details for exclusion criteria although you are using spot urine you need to explain if you are excluding participants with hypertension medications or diuretics medications. these points should include in exclusion even if spot urine is used. 

**For component three, we have collected data on participants' use of medications for hypertension and, specifically, if they are using diuretics. We will assess whether there are differences in urine sodium excretion for those with hypertension or on diuretic therapy. Where necessary, we will exclude participants on diuretic therapy from analyses or conduct sensitivity analyses with models excluding and including persons on diuretic therapy.  For component one, we included hypertension as a covariate in our analyses. A note to this effect has been added to the section on inclusion and exclusion criteria for component three.**

**Note also that the analyses for component one have now been completed and a paper published.**

- Are you using two formulas to estimate sodium intakes **NTERSALT formula and PAHO formula....are aiming to compare between them or you will use one formula and which one fit Jamaica population.

**We will use the PAHO formula as the since this allows us to estimate both sodium and potassium. We have removed the INTERSALT formula from the protocol.**

- Component 2: salt content of foods sold in restaurants. Very good but the aim should be clear. the aim her not to estimate sodium but to identify main source of sodium in food intakes. 

**We have adjusted the aim as follows: To assess the use of sodium in the preparation of restaurant foods and estimate the sodium content in restaurant foods.**

- Component 3, how you will assess KAP which protocol or questionnaire and are you using scoring system just to describe KAP. 

**The analyses for the evaluation of knowledge attitudes and practices will be primarily descriptive, but we will use scores to classify persons in KAP categories. These categories will be created in the analysis phase of the study.**

- Component 4, In order to do it correct you need to include more information and measurements regarding exclusion criteria such as acceptable total 24-hour urine sample 500 ml and above, acceptable level of 24-hour creatinine to measure adequate 24-hour urine sample, measure amount of lean meat intake, these points can be found in studies dealing with 24-hour urine. Including IPAQ very good but not limited to assess physical activity but also important for justify differences in estimated sodium intake between 24-hour and spot urine. 

**We will exclude participants whose collection time is <500 ml and <22 hours or >26 hours; we will also exclude participants whose 24-hour urine creatinine is greater than 2SD above or below the sex-specific mean (Du et al 2021; Jackson et al 2020). We will also assess whether physical activity level is associated with 24-hour or spot urine sodium and adjust accordingly.  We did not collect data on lean meat intake, therefore we have listed this as a limitation.**

- General notes, what are reference for food frequency questionnaire is it general or specific for salt intake (Data collection).

**The FFQ was derived from a local FFQ which was previously validated for use in Jamaica (Jackson, 2011). The FFQ has undergone several updates using 24-hour recalls to calibrate food listings and capture detailed use of sodium and related food preparation practices. Instruments have been used successfully in a sodium reduction trial (Jamaica) and a more recent Barbados study of ultra-processed food (UPF) intakes (Forrester 2005; Harris 2021).**

- it will be good to include intervention studies in references and introduction to highlight benefit of this study for future intervention studies. 

**A paragraph on the effect of interventions to reduce sodium has been added to the introduction.**

# Reviewer report: Reviewer response for version 1

**DOI:** 10.5256/f1000research.134637.r182576

**Published:** 2023-08-31

**Authors:** Adriana Blanco-Metzler

**Affiliations:** 1 Costa Rican Institute of Research and Teaching in Nutrition and Health (INCIENSA), Tres Ríos 4-2250, Costa Rica

**Title:** doesn’t reflect all objectives or components from the protocol (for ex. sodium content in foods is lacking)

**Introduction:**

- Since restaurant foods will exclusively be studied by sodium content, more information is necessary to understand why these were selected instead of home-made foods and/or processed foods.
- Repeats the purpose of the protocol: first, indicates that seeks to conduct base line assessment and later specifies the objectives.

**Methods:**

- Component 1: Sociodemographic and biomedical variables could be listed to have a better idea which were selected.
- Component 2: a) what type of restaurants you plan to select? Do you have a classification of the types of restaurants available in Jamaica? b) How usual is it to know the sodium content of preparations and/or their ingredients in Jamaica? c) What happens if there is not enough and updated information on sodium content of these foods, especially local? Would you use food composition tables to estimate the sodium content in the preparations, which ones? d) How do you plan to do the sensibility analysis (method?) e) If you plan to do food analysis for sodium a protocol for sampling, managing the sample and methodological aspects is needed. f) Do you have a criterion for grouping the specified food group categories?
- Component 3: In addition to age what other variables will you consider when selecting the sample? For ex. Sex, income, studies, with or without hypertension or CVD, etc

Is the study design appropriate for the research question?

Yes

Is the rationale for, and objectives of, the study clearly described?

Yes

Are sufficient details of the methods provided to allow replication by others?

Partly

Are the datasets clearly presented in a useable and accessible format?

Yes

Reviewer Expertise:

Public Health, Food Science and Nutrition

I confirm that I have read this submission and believe that I have an appropriate level of expertise to confirm that it is of an acceptable scientific standard, however I have significant reservations, as outlined above.

# Response

**Published:** 2023-11-22

**Authors:** Trevor S Ferguson

**Affiliations:** The University of the West Indies, Jamaica

To: Adriana Blanco-Metzler

 Thank you for taking the time to review this manuscript and for your insightful recommendations.

 Please see below a point by point response to the specific comments. The responses are in bold type.

**Title:** doesn’t reflect all objectives or components from the protocol (for ex. sodium content in foods is lacking)

 Revised Title: **The Jamaica Salt Consumption Study Protocol: Sodium Intake; Sodium Content in Restaurant Foods; Knowledge, Attitudes, and Practices; Spot Urine Sodium Validation**

**Introduction: **

- Since restaurant foods will exclusively be studied by sodium content, more information is necessary to understand why these were selected instead of home-made foods and/or processed foods.

**The sodium content of pre-packaged foods and beverages, available retail in Jamaica, were assessed previously as part of a separate study (Soares-Wynter 2020).  This has now been added in the introduction**

- Repeats the purpose of the protocol: first, indicates that seeks to conduct base line assessment and later specifies the objectives.

**The first statement was removed and the specific objectives kept.**

**Methods:**

- Component 1: Sociodemographic and biomedical variables could be listed to have a better idea which were selected.

**Supplementary file  with list of variables added. See: https://doi.org/10.6084/m9.figshare.24589452**

- Component 2: a) what type of restaurants you plan to select? **All restaurants serving meals that are ready to eat will be eligible for inclusion**
- Do you have a classification of the types of restaurants available in Jamaica? **There is no standard system of restaurant classification in Jamaica. We have defined chain restaurants as chain restaurants if they have three or more local branches. In our analyses we will further define restaurants as fast food restaurants; standard dining in restaurants and cafes. ** 
- b) How usual is it to know the sodium content of preparations and/or their ingredients in Jamaica? **Sodium content of restaurant foods are usually not known as it is not required for restaurants to have available nutrition information. The purpose of the study is to try to ascertain this by getting information on ingredients used and food preparation practices.**  
- c) What happens if there is not enough and updated information on sodium content of these foods, especially local? Would you use food composition tables to estimate the sodium content in the preparations, which ones? **Yes, where data are not available, we will use publicly available databases such as the USDA Food database. See extract from paragraph 3 in the section from component 2: “We will also seek to obtain information on nutrient content of food sold if these have been previously determined. We will also gather publicly available data on recipes, ingredient quantities, and salt content of meals from restaurant websites and/or social media pages. Where we have difficulty obtaining specific recipes or ingredient quantities, we will estimate salt content based on typical recipes for similar items in the Jamaican context.” We also added the following sentence: “We will also seek to obtain data from sources such as the US Department of Agriculture Food Central Database international chain restaurants.” **
- d) How do you plan to do the sensibility analysis (method?) **This has been removed from the protocol.**  
- e) If you plan to do food analysis for sodium a protocol for sampling, managing the sample and methodological aspects is needed. **This has been removed from the protocol. We will consider food analysis in future studies. ** 
- f) Do you have a criterion for grouping the specified food group categories? **Categories will be created in the analysis stage, guided by the study nutritionist. This has now been added in the revised version.**
- Component 3: In addition to age what other variables will you consider when selecting the sample? For ex. Sex, income, studies, with or without hypertension or CVD, etc. **We employed the Kish Method for sampling within communities. For this procedure, the data collector makes a list of all the household members who are eligible to participate in the study, ordered by age and sex and then select one person based on a random number system. We did not consider hypertension, CVD or other health or social conditions in selecting participants for the study. The text has been updated to reflect this.**
